# Antimicrobial and iNOS inhibitory activities of the endophytic fungi isolated from the mangrove plant *Acanthus ilicifolius var. xiamenensis*

**DOI:** 10.1186/s40529-019-0252-3

**Published:** 2019-03-12

**Authors:** Wei-Chiung Chi, Ka-Lai Pang, Wei-Ling Chen, Guei-Jane Wang, Tzong-Huei Lee

**Affiliations:** 1 Department of Food Science, National Quemoy University, Kinmen, 89250 Taiwan; 2 Institute of Marine Biology, National Taiwan Ocean University, Keelung, 20224 Taiwan; 3 Institutes of Clinical Medical Science and Biomedical Sciences, China Medical University, Taichung, 40402 Taiwan; 4 Department of Medical Research, China Medical University Hospital, Taichung, 40447 Taiwan; 5 Department of Health and Nutrition Biotechnology, Asia University, Taichung, 41354 Taiwan; 6 Institute of Fisheries Science, National Taiwan University, Taipei, 10617 Taiwan

**Keywords:** *Acanthus ilicifolius var. xiamenensis*, Endophytic fungi, Mangrove, Anti-microbial activity, iNOS inhibitory

## Abstract

**Background:**

*Acanthus ilicifolius var. xiamenensis* (Acanthaceae) is an old world mangrove species and has long been used as a folk remedy for treating various ailments in traditional medicine. The nature source of *A. ilicifolius var. xiamenensis* is now in short supply because of the urban development and habitat destruction. To better utilize this resource, biodiversity and bioactivity of endophytic fungi isolated from *A. ilicifolius var. xiamenensis* were investigated.

**Results:**

A total of 168 fungal isolates were cultured from leaves and stems of the mangrove plant collected in January (winter) and July (summer) 2014 at Kinmen County, Taiwan. Spent culture extract of 28 isolates were found to have bioactivities against one of the following pathogenic microorganisms: the bacteria *Bacillus subtilis*, *Staphylococcus aureus* (Gram-positive) and *Escherichia coli* (Gram-negative) and the fungi *Candida albicans* and *Cryptococcus neoformans*. These positive extracts were mostly active against the Gram-positive bacteria and *C. albicans*. *Corynespora cassiicola* NTOU4889 and *Xylaria* sp. NTOU4900 inhibited growth of all 3 test bacteria whereas *Phellinus noxius* NTOU4917 inhibited both test fungi. A further anti-inflammatory study of culture extracts of these 28 isolates revealed that extracts with a high iNOS inhibition caused a low viability of cells, and those with a low iNOS inhibition had a high cell viability. Three extracts showed low cytotoxicity (i.e. > 100% cell viability) and high iNOS inhibition (< 15% of NO production) of cells and they were *Phoma* sp. 2 NTOU4338, *Nodulisporium* sp. NTOU4868 and *Guignardia* sp. NTOU4871.

**Conclusion:**

These results indicate that the endophytic fungi associated with *A. ilicifolius var. xiamenensis* can be a potential source of novel natural active substance.

## Background

With the emergence of antibiotic-resistant bacteria, finding new antibiotic drugs is in dire need (Strobel 2002; Guo et al. 2008). Over the years, medicinal plants have been explored for bioactive substances with anti-bacterial, anti-fungal, anti-cancer and/or anti-viral activities, for example, Paclitaxel (generic name Taxol) (Lin et al. 2007). Paclitaxel, the most famous natural-sourced cancer drug in the world, is derived from the bark of the Pacific yew tree (*Taxus brevifolia*) and is used to treat ovarian, breast, lung, pancreatic and other cancers. Stierle et al. (1993) isolated the fungus *Taxomyces andreanae* from *T. brevifolia*, which was found to produce taxol and this discovery provided a more feasible and practical way to mass-produce this compound. As an increasing number of endophytic fungi with novel metabolites of pharmaceutical importance has been isolated from medicinal plants, these plants may serve as a reservoir of untold numbers of endophytic microorganisms capable of synthesizing bioactive compounds that may act against plant pathogens (Cui et al. 2011). In the past, soil-borne microorganisms were the major sources of medicinal compounds. Currently, endophytic fungi from medicinal plants have been one of the main targets for drug leads, and many undescribed endophytic fungal species may be the sources of new medicines (Huang et al. 2001; Guo et al. 2008; Cui et al. 2011).

Around 50–70 species of mangrove plants are distributed in tropical and subtropical climates in the world (FAO 2007). Several mangrove plants have been studied for their endophytic fungal association, such as *A. ilicifolius*, *Aegiceras corniculatum*, *Arthrocnemum indicum*, *Avicennia officinalis*, *Av. marina*, *Bruguiera gymnorrhiza*, *Ceriops decandra*, *Excoecaria agallocha*, *Kandelia candel*, *Lumnitzera racemosa*, *Rhizophora apiculata*, *Rh. mucronata*, *Sesuvium portulacastrum*, *Sonneratia caseolaris*, *Suaeda fruticosa* and *Su. maritima* (Fisher and Petrini 1987; Purkayastha and Pal 1996; Suryanarayanan et al. 1998; Suryanarayanan and Kumaresan 2000; Kumaresan and Suryanarayanan 2001; Okane et al. 2001; Ananda and Sridhar 2002). The Ascomycota is dominant with many asexual species while the Basidiomycota is uncommon (Sebastianes et al. 2013).

*Acanthus ilicifolius var. xiamenensis* (Acanthaceae) is an old world mangrove species and characterized by spiny leaves, spicate terminal inflorescences, two bracteoles and uniform anthers (Duke 2006). This plant has long been used as a folk remedy for treating various ailments in traditional medicine (Ragavan et al. 2015; Saranya et al. 2015). Various parts of the plant have been used as crude drugs for treatment of asthma, diabetes, dyspepsia, leprosy, hepatitis, paralysis, snake bite, rheumatoid arthritis and diuretic (Bandaranayake 1998). In Taiwan, the only distribution of *A. ilicifolius var. xiamenensis* is at the Kinmen Island. Little is known on the endophytic fungi associated with this plant and therefore, we initiated a study on the diversity of endophytic fungi of *A. ilicifolius var. xiamenensis* and their anti-microbial and anti-inflammatory activities. In the present study, we report the antimicrobial and iNOS inhibitory activities of the endophytic fungi isolated from leaves and stems of *A. ilicifolius var. xiamenensis*.

## Methods

### Endophytic fungi of *A. ilicifolius var. xiamenensis*

One hundred and sixty-eight isolates of endophytic fungi were isolated from 95 leaves of *A. ilicifolius var. xiamenensis* (5 trees) collected in January and July 2014 and identified based on sequencing of a region of the rDNA spanning from 18S to 28S including ITS1 (internal transcribed spacer 1), ITS2 and 5.8S rDNA and comparing these sequences with those in the GenBank using nucleotide BLAST search. (Chi et al. unpublished results). These fungi were subcultured on malt extract agar (MEA) plates for 1 week (Table 1). Two agar plugs (8 mm in diameter) were made from the growing edge of the colonies and inoculated into 100 ml GYP broth (0.2 g peptone, 1 g dextrose, 0.1 g yeast extract) in 250 ml Erlenmeyer flasks. The flasks were incubated for 14 days at 25 °C on an orbital shaker at 220 rpm/min.

**Table 1:** Anti-microbial and anti-inflammatory activities of spent culture liquid of the 28 out of 168 endophytic fungi isolated from *Acanthus ilicifolius var. xiamenensis*

			Anti-iNOS assay (Griess)		Cell viability assay (Alamar Blue)		Anti-microbial activity				
Taxa **(**GenBank accession number)	Ordinal classification	Isolation season	Mean	S.E.	Mean	S.E.	*Bacillus subtilis*	*Escherichia coli*	*Staphylococcus aureus*	*Candida albicans*	*Cryptococcus neoformans*
*Hortaea werneckii* (MK432978)	Capnodiales	Winter	100.85	4.78	104.86	0.64	+	–	–	–	–
*Zasmidium citri* (MK432986)	Mycosphaerellales	Winter	28.04	1.47	106.17	2.33	+	–	+	–	–
*Colletotrichum* sp. 3 (MK432994)	Glomerellales	Winter	72.59	3.24	101.48	0.63	+	–	+	+	–
*Alternaria alternata* (MK432953)	Pleosporales	Winter	0.00	0.22	0.00	0.00	+	–	+	–	–
*Phoma* sp. 2 (MK432990)	Pleosporales	Winter	14.58	0.82	114.35	6.83	–	–	+	–	–
*Cladosporium* sp. 2 (MK432958)	Capnodiales	Winter	138.82	2.76	100.02	1.96	+	–	–	–	–
*Didymella* sp. (MK432968)	Pleosporales	Winter	66.40	13.22	102.72	1.13	–	+	–	–	–
*Phoma* sp. 2 (MK432991)	Pleosporales	Winter	67.04	1.60	102.14	0.80	–	–	+	–	–
*Colletotrichum* sp. 1 (MK432992)	Glomerellales	Winter	7.35	0.79	89.56	1.68	+	–	+	+	–
*Fusarium* sp. (MK432970)	Hypocreales	Summer	74.57	3.13	99.36	1.47	+	–	–	–	–
*Nodulisporium* sp. (MK432980)	Xylariales	Summer	0.00	3.01	105.59	3.71	+	–	+	–	–
*Guignardia* sp. (MK432976)	Botryosphaeriales	Summer	12.88	0.62	104.09	1.45	–	–	+	–	–
*Guignardia* sp. (MK432977)	Botryosphaeriales	Summer	44.70	0.68	28.63	2.50	–	–	+	–	–
*Fusarium* sp. (MK432971)	Hypocreales	Summer	85.10	2.41	92.82	0.60	+	–	–	–	–
*Fusarium* sp. (MK432972)	Hypocreales	Summer	71.54	9.82	103.85	2.33	+	–	–	–	–
*Didymella* sp. (MK432969)	Pleosporales	Summer	2.27	0.48	77.52	2.41	–	+	–	–	–
*Cladosporium* sp. 2 (MK432959)	Capnodiales	Summer	95.49	2.17	93.57	2.06	+	–	–	–	–
*Corynespora cassiicola* (MK432963)	Pleosporales	Summer	1.81	1.28	62.52	1.21	+	+	+	–	–
*Pseudocercospora nymphaeacea* (MK432983)	Mycosphaerellales	Summer	3.06	0.18	3.18	4.66	+	–	+	–	–
*Xylaria* sp. (MK432985)	Xylariales	Summer	84.00	2.57	96.33	0.99	+	+	+	–	–
*Colletotrichum* sp. 3 (MK432996)	Glomerellales	Summer	90.86	4.55	94.27	1.41	+	–	+	+	–
*Phanerina mellea* (MK432982)	Polyporales	Summer	62.25	4.45	93.68	0.52	+	–	+	–	–
*Aureobasidium* sp. 2 (MK432957)	Dothideales	Summer	69.34	1.61	104.48	5.97	+	–	–	–	–
*Pestalotiopsis microspora* (MK432981)	Amphisphaeriales	Summer	48.94	1.40	98.28	3.44	–	–	+	–	–
*Tinctoporellus epimiltinus* (MK432984)	Polyporales	Summer	53.50	2.22	92.61	0.73	+	–	+	–	+
*Phellinus noxius* (MK440618)	Hymenochaetales	Summer	155.02	2.58	108.85	0.66	–	–	+	+	+
*Phomopsis* sp. 2 (MK432997)	Diaporthales	Summer	31.82	2.36	101.55	3.62	+	–	–	+	–
*Diaporthe endophytica* (MK432966)	Diaporthales	Summer	72.54	2.00	101.96	1.23	+	–	–	–	+

+ = with activity, – = without activity; S.E. = standard error

### Secondary metabolite extraction

After incubation, the mycelia were separated from the spent culture broth by filtration. The filtered broth was partitioned two times with an equal volume of recycled ethyl acetate (AcOEt) and concentrated in vacuum to dryness. The solid AcOEt extracts were dissolved in sterilized water to a final concentration of 0.5 mg/ml for the antimicrobial assays described below.

### Test indicator organism

The test indicator bacteria included Gram-negative *Escherichia coli* and two Gram-positive *Bacillus subtilis* and *Staphylococcus aureus*, all available at the Institute of Fisheries Science, National Taiwan University. All bacteria were cultured in Luria broth (LB) at 37 °C for 18 h and maintained on LB agar. Two test indicator fungi, namely *Candida albicans* and *Cryptococcus neoformans*, obtained from Faculty of Kinmen County Health Bureau, were used in the study. Both fungi were cultured in Yeast Mold (YM) broth at 30 °C for 48 h and maintained on YM agar.

### Anti-microbial assay

The agar well diffusion method was used to evaluate antimicrobial activity of the spent culture broth of the endophytic fungi (Rios et al. 1988; Cui et al. 2011). The bacteria were diluted using beef extract peptone (BEP) medium (5 g beef extract, 5 g NaCl, 10 g peptone, 15 g agar, 1 L of sterilized water) to give a concentration of 1 × 10^6^ bacteria/ml and poured into a Petri dish (9 cm diameter) containing BEP agar medium. Test fungi were spread evenly on the surface of YM agar plates, and incubated for 3–5 days at 25 °C. Cell concentrations of the test fungi were diluted using molten Sabouraud agar (SA) medium to 1 × 10^5^ spores/ml, and 10 ml of this diluted medium were poured into a Petri dish containing 8 ml of solidified SA medium. A flame-sterilised cork borer was used to make circular wells (7.8 mm in diameter) in the BEP and SA agar. Fungal extracts (40 μl) with a final concentration of 0.5 mg/ml were added into the wells. After incubation at 37 °C for 24 h and at 25 °C for 48 h for bacteria and fungi, respectively, antibacterial and antifungal activities were measured in terms of diameter of the inhibition zone in triplicates.

### Anti-inflammatory assay

Nitrite production and cell viability assay were used to assess the effects of the fungal extracts on LPS-induced NO production (Wang et al. 2007). The ethyl acetate soluble fraction, two positive controls [Nω-nitro-l-arginine (L-NNA, a non-selective iNOS inhibitor) and aminoguanidine (a specific inhibitor of iNOS)] and the vehicle (0.1%, DMSO) were added to the RAW 264.7 cells in the presence of LPS (200 ng/mL). Nitrite formation and cell viability were measured with the Griess reagent and the redox indicator Alamar Blue, respectively (Wang et al. 2007).

## Results

### Antimicrobial activity

**Fig. 1:**
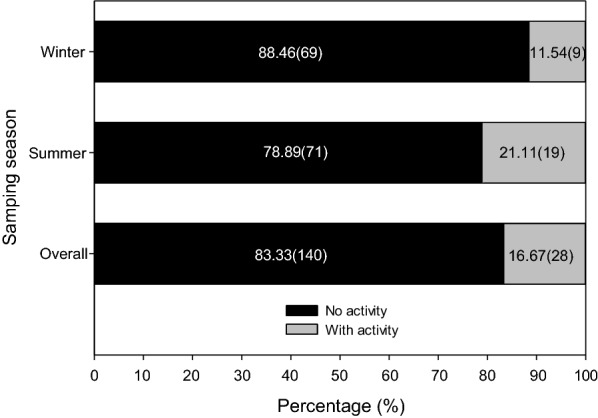
Percentage of endophytic fungi isolated from *Acanthus ilicifolius var. xiamenensis* in winter (January) and summer (July) 2014 with antimicrobial activity

Spent culture extract of the 168 endophytic fungi isolated from *A. ilicifolius var. xiamenensis* was tested for their antimicrobial and anti-inflammatory activities. A total of 28 isolates (16.67%) of the tested endophytic fungi showed antimicrobial activities to at least one indicator organism (Table 1). Within each season, 9 (11.54%) active isolates out of 78 isolates were cultured from winter and 19 (21.11%) out of 90 isolates from summer (Fig. 1). Seven isolates were active against three indicator organisms, 7 against two indicator organisms and 14 against one indicator organisms. No extracts were able to act against all test bacteria and fungi.

**Fig. 2:**
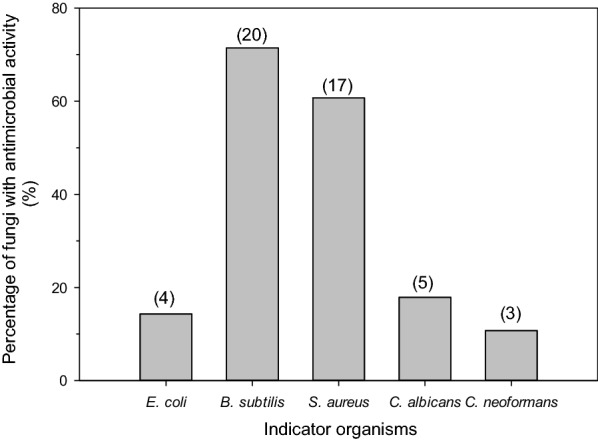
Number and percentage of endophytic fungi isolated from *Acanthus ilicifolius var. xiamenensis* against *Escherichia coli*, *Bacillus subtilis*, *Staphylococcus aureus*, *Candida albicans* and *Cryptococcus neoformans*

Twenty (11.90%) and seventeen (10.12%) extracts were active against the Gram-positive *B. subtilis* and *S. aureus*, respectively (Fig. 2). However, only 4 (2.38%) extracts were active against the Gram-negative bacterium *E. coli*. Few extracts were active against the test fungal pathogens; 5 (2.98%) and 3 (1.79%) extracts showed anti-fungal activity against *C. albicans* and *C. neoformans*, respectively.

Concerning the isolates affiliated with the Ascomycota, the Xylariales (*Nodulisporium* sp. NTOU4868 and *Xylaria* sp. NTOU4900) were only active against the tested bacteria but not the fungal pathogens, and the same is true for the Amphisphaeriales, the Botryosphaeriales, the Capnodiales, the Dothideales, the Hypocreales, the Mycosphaerellales and the Pleosporales (Table 1). Most extracts were active against the Gram-positive bacteria, but only the extracts of *Didymella* sp. NTOU4354 and NTOU4878, *Corynespora cassiicola* NTOU4889 and *Xylaria* sp. NTOU4900 were active against *E. coli*. Only the ascomycetous orders Diaporthales, Glomerellales, the basidiomycetous orders Hymenochaetales and Polyporales were able to inhibit growth of the pathogenic fungi, especially *C. albican*. Growth of *C. neoformans* were only inhibited by extracts of *Diaporthe endophytica* NTOU4920 (Diaporthales), *Phellinus noxius* NTOU4917 (Hymenochaetales) and *Tinctoporellus epimiltinus* NTOU4916 (Polyporales). For *C. albicans*, the extracts of *Colletotrichum* spp., *Phellinus noxius* NTOU4917 and *Phomopsis* sp. 2 NTOU4918 were active against it.

### Anti-inflammatory activity

**Fig. 3:**
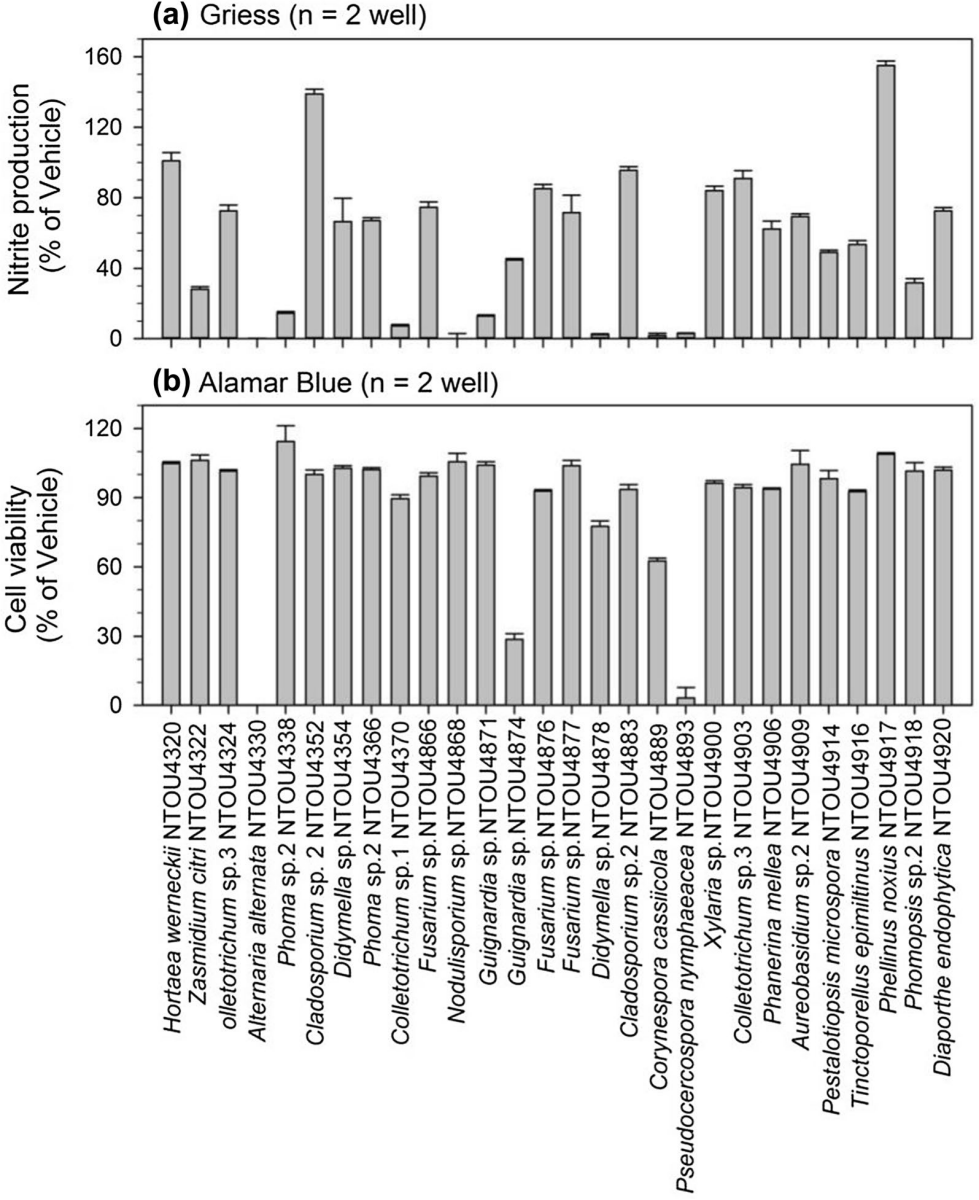
Results of **a** the anti-iNOS (Griess) and **b** the cell viability assays (Alamar Blue)

The 28 isolates with anti-microbial activity were further tested for their anti-inflammatory activity through Griess (nitric oxide production) and Alamar Blue (cell viability) assays and the results are shown in Table 1 and Fig. 3. The highest value (% of vehicle) of Griess assay was 155.02 (*Phellinus noxius* NTOU4917), followed by 138.82 (*Cladosporium* sp. 2 NTOU4352), whereas the lowest was *Alternaria alternata* NTOU4330 and *Nodulisporium* sp. NTOU4868 (Fig. 3a). The values (% of vehicle) of Alamar Blue assay ranged from 0 (*Alternaria alternata* NTOU4330) to 114.35 (*Phoma* sp. 2 NTOU4338) (Fig. 3b). Generally, extracts with high iNOS inhibition (i.e. low NO production) also caused low viability of cells, except *Nodulisporium* sp. NTOU4868 and the reverse is true, i.e. low iNOS inhibition and high cell viability. Low (< 10%,  % of vehicle) nitric oxide (NO) production was found for cells treated with extracts of six isolates: *Alternaria alternata* NTOU4330, *Nodulisporium* sp. NTOU4868, *Corynespora cassiicola* NTOU4889, *Didymella* sp. NTOU4878, *Pseudocercospora nymphaeaceae* NTOU4893 and *Colletotrichum* sp. 1 NTOU4370; no NO production was in fact produced by the first two isolates (Fig. 3a). On the other hand, high (> 100%,  % of vehicle) NO production was recorded from cells of *Hortaea werneckii* NTOU4320, *Cladosporium* sp. 2 NTOU4352 and *Phellinus noxius* NTOU4917. For cell viability, 23 of the 28 test extracts showed high cell viability (> 89.56%, % of vehicle) (Table 1, Fig. 3b). No cells were viable when they were treated with extract of *Alternaria alternata* NTOU4330. Low cell viability (3.18%) was also observed in cells treated with extract of *Pseudocercospora nymphaeacea* NTOU4893. Three extracts showed low cytotoxicity (i.e. > 100% cell viability) and high iNOS inhibition (< 15% of NO production) of cells and they were *Phoma* sp. 2 NTOU4338, *Nodulisporium* sp. NTOU4868 and *Guignardia* sp. NTOU4871.

## Discussion

This is the first report that fungal endophytes associated with *A. ilicifolius var. xiamenensis* at Kinmen have been found to possess the potential of the iNOS inhibitory and antimicrobial activity. *Acanthus ilicifolius var. xiamenensis* and its congeners are widely used in traditional medicine (Saranya et al. 2015). *Acanthus montanus* is used in the treatment of diseases such as backache, cough, rheumatic pains and chest pain (Adeyemi et al. 2005), and pain, female infertility (Thierry et al. 2011). The methanolic fraction of *A*. *ilicifolius* leaf extract produced significant inhibition of rat paw oedema (Adeyemi et al. 2005). Endophytic fungi isolated from *Acanthus* have also been shown to have anti-microbial properties (Maria et al. 2005; Chen et al. 2007). In this study, anti-microbial and anti-inflammatory properties of spent culture extracts of 168 endophytic fungi isolated from surface-sterilised leaves and stems of *A. ilicifolius var. xiamenensis* were examined. These extracts were mostly active against the Gram-positive *B. subtilis* (20 active extracts) and *S. aureus* (17), in contrast to only 4 active extracts against the Gram-negative bacterium *E. coli*. These results are in agreement with reports of anti-bacterial activity of endophytic fungi from mangrove plants showing stronger activities against Gram-positive over Gram-negative bacteria (Chareprasert et al. 2010; Ebrahimia et al. 2010). This difference of anti-bacterial activity between Gram-positive and Gram-negative bacteria might be due to their differences in cell wall composition. The outer layer of cell wall of Gram-negative bacteria composes of lipopolysaccharide, in contrast to a thick layer of peptidoglycan in Gram-positive bacteria. Ethyl acetate extracts comparatively less polar natural products than other solvents such as methanol (Borquaye et al. 2016), suggesting that less polar natural products might be responsible for the anti-microbial activity of the majority of the extracts observed against Gram-positive bacteria in this study. *Corynespora cassiicola* NTOU4889 and *Xylaria* sp. NTOU4900 showed activity to all test pathogenic bacteria in this study. An endophytic *Xylaria* sp. from the mangrove *A. ilicifolius* in Thailand also had anti-bacterial properties against both Gram-positive and Gram-negative bacteria (Chareprasert et al. 2010). The Xylariales is known to produce anti-bacterial compounds (Xu et al. 2015).

Generally, the extracts of the spent culture of the isolated fungi showed weak anti-fungal activity towards the two test fungal pathogens. Two crude extracts out of six fungi isolated from *A. ilicifolius var. xiamenensis* using submerged fermentation showed anti-fungal activity against *C. albicans*, while anti-fungal activity against *C. albidus* was shown after these extracts were partially purified and they suggested a possible interference between different active principles in the crude extracts. Whether the same reason caused a weak anti-fungal activity by the fungal extracts in this study requires further studies (Maria et al. 2005).

Crude extracts of many fungi in this study showed comparable high iNOS inhibition activity but some also with high cell toxicity. Three crude extracts (*Phoma* sp. 2 NTOU4338, *Nodulisporium* sp. NTOU4868 and *Guignardia* sp. NTOU4871) showed high iNOS inhibition and low cytotoxicity and these extracts should be fractionated to determine the active chemical constituents. In a similar research, Chen et al. (2017) reported that culture extracts of the endophytic fungus *Lasiodiplodia theobromae* isolated from *A. ilicifoliu*s showed anti-inflammatory activities and they isolated some active components from the extracts including Lasiodiplactone A, an unprecedented lactone that possesses a unique tetracyclic system. Compounds such as meroterpenoid identified from *Aspergillus terreus* H010, an endophytic fungus from *Kandelia obovata*, also exhibited anti-inflammatory activities (Liu et al. 2017). Results from these reports and those from our study strongly support the view that endophytic fungi of mangrove plants are promising sources of natural bioactive compounds (Stierle et al. 1993; Strobel et al. 1997; Strobel and Daisy 2003; Chen et al. 2016).

**Fig. 4:**
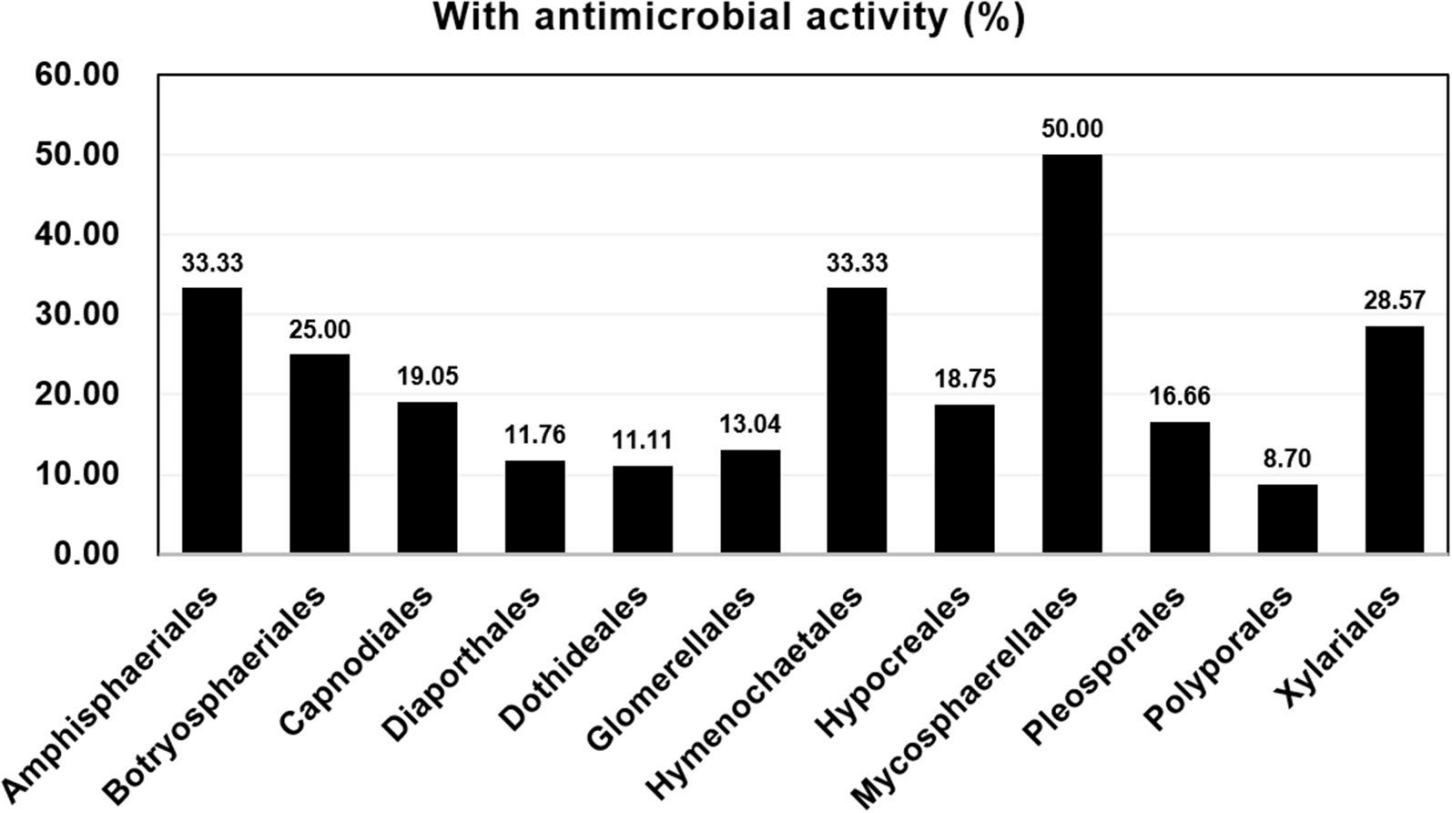
Proportion of the ordinal classification of endophytic fungi isolated from *Acanthus ilicifolius var. xiamenensis* with anti-microbial activity

Figure 4 shows the percentage of fungi (at the ordinal level) with antimicrobial activities, calculated from the original 168 cultures isolated from leaves of *A. ilicifolius var. xiamenensis*. The highest percentages were found in Mycosphaerellales (50.00%), following by Amphisphaeriales (33.33%), Xylariales (28.57%) (Ascomycota) and Hymenochaetales (33.33%) (Basidiomycota); the lowest in Diaporthales (11.76%), Dothideales (11.11%) (Ascomycota) and Polyporales (8.70%) (Basidiomycota). Future screening of bioactive substances from endophytic fungi of mangrove plants can focus on the four orders of fungi with a higher likelihood of finding fungi with anti-microbial and anti-inflammatory activities. However, Moron et al. (2018) found that culture extracts of taxa of the Hypocreales (*Fusarium*, *Trichoderma*) isolated from roots of mangrove plants produced good antimicrobial activities against Gram-positive bacteria. In another study, Handayani et al. (2017) found that culture extracts of the endophytic Eurotiales (*Aspergillus* spp.) isolated from *Sonneratia grifithii* produced good antimicrobial activities against *S. aureus* and *E. coli*.

In this study, 28 out of 168 endophytic isolates of fungi cultured from leaves and stems of *A. ilicifolius var. xiamenensis* were found to have various levels of anti-microbial and anti-inflammatory activities, which may be of pharmaceutical potentials. Endophytes are omnipresent in plants, with diversity dependent on host species and location (Tan and Zou 2001). Endophytic fungi of mangrove plants may include one to several taxa that are only adapted to each of these plant species (Schulz and Boyle 2005). The largest area and highest diversity of mangrove region are found in Asia (FAO 2007), suggesting that there is a high diversity of endophytic fungi in Asia, representing an untapped resource for bioactivity screening (Huang et al. 2014).

## Conclusions

The present study demonstrates that the mangrove plant support wide spectrum of endophytes with significant bioactive potential. Thus, concerted efforts should be carried out for bioprospection in the *A. ilicifolius var. xiamenensis* to tap and conserve the microbial resources of this important biodiversity and utilize their potential for human welfare.
